# Integrating genomic and Tn-Seq data to identify common *in vivo* fitness mechanisms across multiple bacterial species

**DOI:** 10.1128/mbio.01988-25

**Published:** 2025-09-22

**Authors:** Derrick E. Fouts, Thomas H. Clarke, Geoffrey B. Severin, Aric N. Brown, Elizabeth N. Ottosen, Caitlyn L. Holmes, Bridget S. Moricz, Sophia Mason, Ritam Sinha, Mark T. Anderson, Victor DiRita, Michael A. Bachman, Harry L. T. Mobley

**Affiliations:** 1 The J. Craig Venter Institute, Rockville, Maryland, USA; 2 Department of Microbiology and Immunology, University of Michigan Medical School 12266 , Ann Arbor, Michigan, USA; 3 Department of Microbiology and Molecular Genetics, Michigan State University College of Natural Sciences 3078, East Lansing, Michigan, USA; 4 Department of Pathology, University of Michigan Medical School 12266 , Ann Arbor, Michigan, USA; University of California, Berkeley, Berkeley, California, USA

**Keywords:** bacteremia, Enterobacterales, fitness genes, multi-species core genome, Tn-Seq

## Abstract

Sepsis, a life-threatening organ dysfunction, is due to an unregulated immune response to infection. Bacteremia is a leading cause of sepsis, and members of the Enterobacterales cause nearly half of bacteremia cases annually. Although previous Tn-Seq studies identified novel bacteremia-fitness genes, evidence for common pathways across species is lacking. To identify common fitness pathways in five bacteremia-causing Enterobacterales species, we utilized our pan-genome pipeline to integrate Tn-Seq fitness data with multiple available functional data types. Core genes from species pan-genomes were used to construct a multi-species core pan-genome, producing 2,850 core gene clusters found in four of five species. Integration of Tn-Seq fitness data identified 373 protein clusters conserved in all five species and a fitness gene in at least one of them. A scoring rubric was applied to these clusters, which incorporated Tn-Seq fitness defects, operon localization, and antibiotic susceptibility data, which reduced the number of bacteremia-fitness genes and identified seven common fitness mechanisms. Independent mutational validation of one prioritized fitness gene, *tatC,* showed reduced fitness *in vivo* across all species tested and increased susceptibility to β-lactams that was restored following *tatC* complementation *in trans*. By integrating known operon structures and antibiotic susceptibility with Tn-Seq fitness data, common genes within the core pan-genome emerged and revealed mechanisms essential for survival in the mammalian bloodstream. Our prediction and validation of *tatC* as a common bacteremia fitness factor supports the utility of this bioinformatic approach. This study represents a major step forward in prioritizing novel targets for therapy against sepsis infections.

**IMPORTANCE:**

Bacteremia is a leading cause of sepsis, a life-threatening condition where an unregulated immune response to infection causes systemic organ failure. Nearly half of bacteremia cases are caused by members of the Gram-negative bacterial taxonomic order Enterobacterales. Given the public health impact of bacteremia and the reduction of existing antibiotic treatment options, novel strategies are needed to combat these infections. In this study, pan-genome software was used to predict seven shared fitness pathways in these bacteria that may serve as novel targets for the treatment of bacteremia. Briefly, a scoring rubric was applied to shared pan-genome clusters, which incorporated multiple data types, including Tn-Seq fitness defects, operon localization, and antibiotic susceptibility data to rank and prioritize fitness genes. To validate one of our predictions, mutations were constructed in *tatC*, which showed both reduced fitness in mice in all species tested and increased susceptibility to β-lactam antibiotics; complementation restored fitness and antibiotic susceptibility to wild-type levels. This study takes a novel bioinformatics approach to build a core pan-genome across multiple distantly related bacteria to integrate computational and experimental data to predict important shared fitness genes and represents a major step forward toward identifying novel targets of therapy against these deadly, widespread, life-threatening infections.

## INTRODUCTION

Sepsis is the life-threatening organ dysfunction resulting from a dysregulated and overwhelming immune response to infection. There are an estimated 49 million cases of sepsis that occur annually worldwide (1), and in the United States, sepsis accounts for one in every 2−3 hospital deaths (2). Bacteremia, or the presence of bacteria in the bloodstream, is the leading cause of sepsis, with Gram-negative species belonging to the Enterobacterales order among the most common causes of bacteremia (3–5). Drug resistance among this cohort of pathogens is a major limitation to effective treatment (4, 6) and contributes to the large burden of antibiotic-resistant bacterial threats recognized by the Centers for Disease Control and Prevention (7). Given the public health impact of Enterobacterales bacteremia and the waning efficacy of existing treatments, novel strategies are needed to combat these infections. To develop such approaches, it is critical to first understand the genetic and molecular basis by which these bacteria cause bloodstream infections (BSIs).

Bacteremia can be divided into three phases of pathogenesis: initial primary site infection, dissemination to the bloodstream, and growth and survival in blood and filtering organs (8). The primary site can also serve as a reservoir for the pathogen that intermittently re-seeds the bloodstream and prolongs systemic infection. We have previously determined that Enterobacterales species replicate rapidly in the liver and spleen during bacteremia (9). For some tissue and species combinations, bacterial burdens decrease over time, reflecting an ability of the host immune system to overcome this rapid proliferation, whereas in other instances, bacterial growth outpaces clearance and bacterial numbers rapidly expand. This balance between a bacterium’s ability to proliferate and withstand immune-mediated clearance can be collectively described as infection fitness.

To define the genetic requirements for “bacteremia-fitness,” we previously performed a series of transposon sequencing (Tn-Seq) studies in which bacterial genes disrupted by transposon insertion mutations are assessed for their contribution to bacteremia-fitness (10–13). These studies have resulted in the identification and characterization of fitness genes ranked by impact for four Enterobacterales BSI pathogens: *Escherichia coli*, *Klebsiella pneumoniae*, *Citrobacter freundii*, and *Serratia marcescens*. Fitness gene identification has also allowed us to build models of metabolism and characterize bacterial pathways required for replication and survival in the host, enabling opportunities for the development of novel therapeutic strategies against these antibiotic-resistant pathogens. Although the Tn-Seq studies have provided valuable insight on an organismal level, comprehensive information on shared and unique fitness strategies across Gram-negative species is lacking, due in part to the unique nature and depth of each transposon insertion library. By integrating Tn-Seq fitness data sets with comprehensive pan-genomic information on gene content, operon structure, and gene function across species, we can gain a better understanding of infection processes for this important group of pathogens.

Our pan-genome pipeline (14) uses PanOCT (15) to cluster orthologous proteins to identify core, variable, and singleton gene or protein clusters and provides the data structure to easily link gene functions, such as GO terms and antimicrobial resistance (AMR) genes, as well as linkage of genes into genomic islands. The core genome of a bacterial pathogen is the collection of genes shared by all or nearly all strains of a species. This core genome is generally enriched for critical functions, including energy production, amino acid metabolism, metabolite transport, nucleotide metabolism, and translational machinery (16). On the other hand, the accessory genome is the set of genes that vary across strains of a species. These genes are often involved in protein secretion and defense against innate host immunity, as well as many niche-specific functions that include traits such as fimbriae, toxins, or iron acquisition systems, which reside in so-called “flexible genomic islands” or fGIs (17).

In this report, we first constructed individual species pan-genomes to identify genes shared within *E. coli, K. pneumoniae, C. freundii, S. marcescens*, and *Enterobacter hormaechei* genomes. Each species’ pan-genome represents the sum of the core and accessory genes for all strains of a species under study. To add biological context, known operon structures, virulence gene predictions, AMR genes, and Tn-Seq bacteremia-fitness data were overlaid onto each pan-genome, visually depicted by using the program *PanACEA* (18). Then, we took each species’ core genes to build a multi-species core pan-genome, which identified the core genes shared across species (19). By integrating our multi-species pan-genome and genome-wide Tn-Seq fitness data, we were able to infer fitness genes in *E. hormaechei,* which lacked Tn-Seq data. We identified 373 protein clusters that were conserved in all five species and predicted to contribute to bacteremia in at least one of them. Applying a scoring rubric to these bacteremia protein clusters, which incorporated the magnitude of a cluster’s fitness defect as predicted by Tn-Seq, its operon localization, and published antibiotic susceptibility data, seven common bacteremia-fitness pathways were identified. Finally, to validate our findings within the prioritized list of bacteremia-fitness genes, the twin-arginine translocation (Tat) system was selected to test independent mutations of four bacteremia-causing *Enterobacterales* for reduced fitness *in vivo* and for increased susceptibility to β-lactam antibiotics *in vitro*. The TatC transmembrane protein strongly contributes to *C. freundii* bacteremia fitness (11) and was likewise predicted to be a bacteremia-fitness factor in *K. pneumoniae* (13), but not in the *S. marcescens* (10, 20), UPEC *E. coli* (12), or *E. hormaechei*, which lacked Tn-Seq data. Independent insertional inactivation of *tatC* in *E. coli*, *K. pneumoniae*, *S. marcescens,* and *E. hormaechei* showed reduced fitness *in vivo*, confirming one of our bioinformatics fitness predictions, even in the three species lacking Tn-Seq evidence. Consistent with predictions from *E. coli*, the *tatC* mutants of the other four species also showed increased susceptibility to β-lactam and β-lactam plus inhibitor that was restored following *tatC* complementation *in trans*. This raises the exciting possibility that inhibitors of these gene products could simultaneously decrease *in vivo* fitness and sensitize the bacteria to FDA-approved standard-of-care antibiotics. Combined, this study provides an atlas of gene conservation across the *Enterobacterales* and the fitness contribution of these genes to bacteremia, a powerful resource that can be leveraged to advance our understanding of pathogenesis and explore novel therapeutic targets.

## RESULTS

### Species pan-genomes

**Fig 1:**
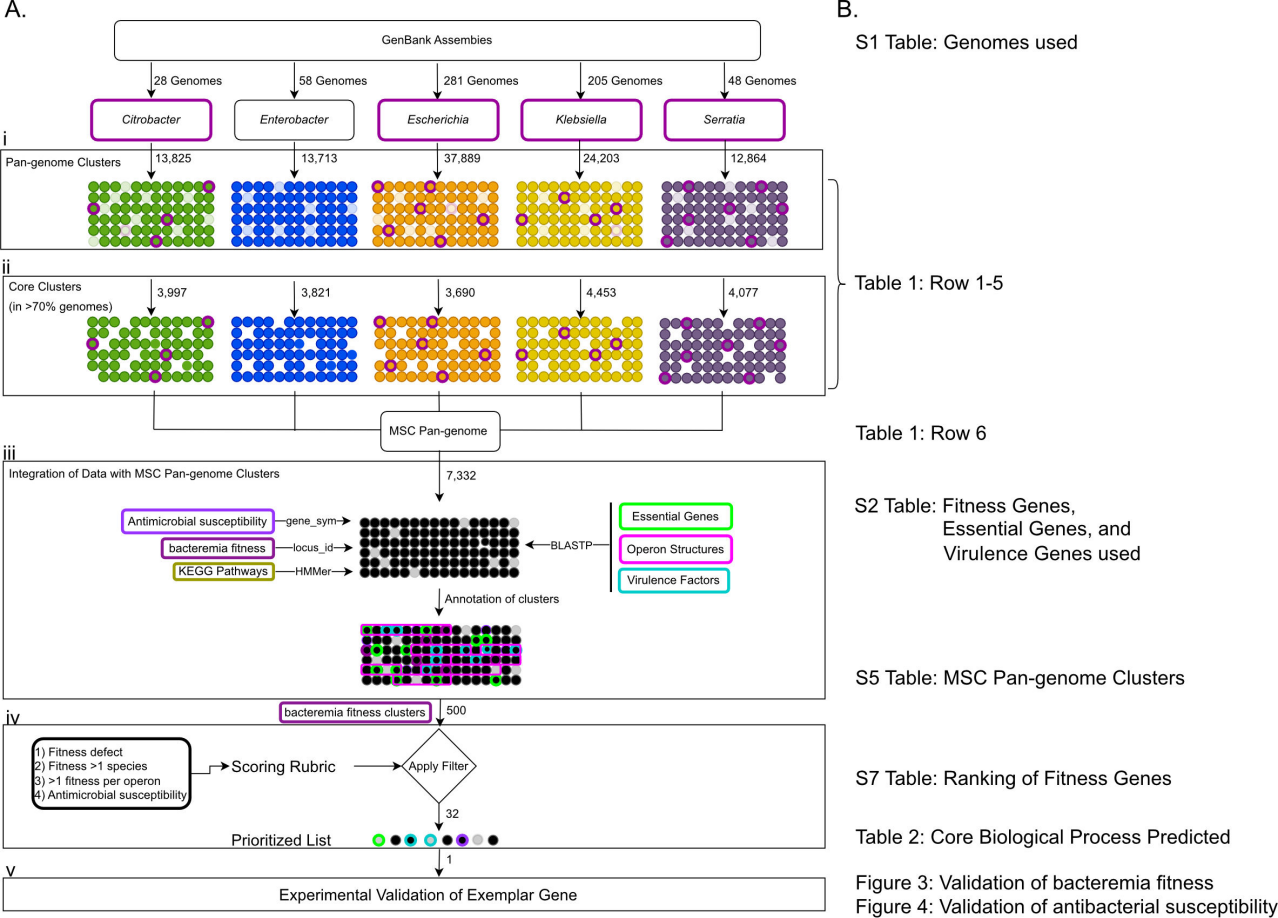
Construction of single and multi-species core pan-genomes. The workflow of bioinformatic processes used in this study is depicted (**A**). Genome assemblies for the five genera shown were downloaded from the NCBI, including those genomes used in Tn-Seq studies to identify fitness genes (purple outline) (10–13). The assemblies were used as input to the JCVI pan-genome pipeline to create pan-genomes for each species (i). From the resulting pan-genome clusters, core proteins (orthologs present in >70% of the genomes) were selected (ii) as input for the multi-species core pan-genome using the same pipeline. In addition to the TIGRFam and CARD annotations from the pan-genome pipeline, we integrated six annotation features from multiple sources into the resulting MSC pan-genome clusters (iii). *E. coli* essential genes (23–27), operon membership (28, 29), and virulence factor information (21) were assigned through BLASTP, KEGG pathway information was mapped using HMMer, and antibiotic susceptibility (30, 31), and Tn-Seq bacteremia fitness genes were mapped by gene_syms and locus_ids, respectively. To only those MSC pan-genome clusters labeled as Tn-Seq bacteremia fitness, we applied a scoring rubric to filter the list of 500 proteins down to 32 prioritized proteins (iv). Finally, an exemplar gene, *tatC*, was selected for validation of fitness *in vivo* and antimicrobial susceptibility *in vitro* in all five genera (v). The location of the input and output data tables is also shown (**B**).

Protein-level pan-genomes of five common sepsis-causing Enterobacterales species were constructed for two key reasons. First, to integrate multiple data types and Tn-Seq data sets to decipher common bacteremia-fitness factors across species. Second, to generate core pan-genomes that can be used downstream to build a “Multi-Species Core” (MSC) pan-genome that represents highly conserved genes that may be targets of interest for future therapies that would treat infections from multiple species (Fig. 1). Proteins from representative genomes of *E. coli*, *K. pneumoniae*, *S. marcescens, C. freundii,* and *E. hormaechei* were used to first build species pan-genomes using PanOCT separately within the JCVI pan-genome pipeline (Table S1) (see S1–S5 Data sets at https://doi.org/10.5281/zenodo.15793688). Centroids (i.e., orthologous protein cluster representatives) of core and accessory pan-genome clusters were searched against curated databases of bacteremia-fitness genes as defined by transposon insertion-site sequencing, classic *E. coli* “essential” genes, virulence genes (21) (Table S2), and AMR genes (22), and the results are summarized in Table 1.

**TABLE 1:** Pan-genome statistics^*f*^

Pan-genome	No. of genomes	No. of clusters	No. of paralogs (no. of clusters; fitness genes)^*d*^	No. of core proteins	No. of accessory proteins	No. of singleton proteins	No. of core pan-chrom asmbls (cycle; chain)^*c*^	No. of fGIs (cycle; chain)	No. of bacteremia-fitness genes^*d*^ (core; accessory)	No. of essential genes^*e*^ (core; accessory)	No. of VFDB genes^*e*^ (core; accessory)	No. of AMR genes (core; accessory)
*C. freundii^a^*	28	13,825	698 (2,743; 3)	3,997	5,211	4,617	4 (2; 2)	2,336 (7; 2,329)	172 (90; 82)	659 (630; 29)	213 (140; 73)	142 (66; 76)
*E. hormaechei^a^*	58	13,713	770 (2,730; NA)	3,821	5,601	4,291	1 (1; 0)	2,971 (13; 2,958)	NA	605 (583; 22)	239 (130; 109)	140 (62; 78)
*E. coli/Shigella* spp.^*a*^	281	37,889	3,279 (19,551; 57)	3,690	14,899	19,300	3 (1; 2)	13,438 (21; 13,417)	233 (117; 116)	755 (677; 78)	1,575 (158; 1475)	160 (67; 93)
*K. pneumoniae^a^*	205	24,203	1,788 (8,006; 2)	4,453	4,970	14,780	1 (1; 0)	7,232 (20; 7,212)	56 (33; 23)	600 (573; 27)	492 (159; 358)	171 (68; 103)
*S. marcescens^a^*	48	12,874	529 (1,705; 6)	4,077	4,984	3,813	3 (0; 3)	2,922 (6; 2,916)	201 (117; 84)	466 (459; 7)	192 (102; 90)	96 (56; 40)
Multi-species core (70% cutoff)^*a*^	5	7,332	155 (35; 20)	2,850	1,369	3,113	49 (0; 49)	1,649 (0; 1,649)	500 (422; 78)	571 (502; 69)	272 (122; 150)	87 (38; 49)
Multi-species core (100% cutoff)^*b*^	5	7,332	ND	2,232	5,100	3,113	ND	ND	373	455	55	27

^*a*^ 70% core definition.

^*b*^ 100% core definition.

^*c*^ cycle, a circular pan-chromosome; chain, a linear pan-chromosome.

^*d*^ from Tn-Seq studies.

^*e*^ Using BLASTP matches with a percent id of >70% and E-value <1×10^−5^.

^*f*^ NA, not applicable; ND, not determined.

The core genomes of each species varied from 3,690 protein clusters in *E. coli* to 4,453 in *K. pneumoniae*. Based on 281 genomes, *E. coli* had the largest pan-genome, encoding 37,889 protein clusters, which is eight times the number of proteins encoded by the average *E. coli* genome (i.e., 4,661 proteins) (Table 1; Table S1). *K. pneumoniae* had the second largest pan-genome with 24,203 protein clusters across 205 genomes, but the number of proteins of this pan-genome was 4.5 times that of the average *K. pneumoniae* genome (i.e., 5,322 proteins). The remaining species' pan-genome number of proteins was between 2.7 and 3.0 times larger than their respective genome averages. The number of strain-specific protein clusters from the species pan-genomes ranged from a minimum of 0 to a maximum of 488 (*Escherichia*: 0−488, *Klebsiella*: 1−296, *Enterobacter*: 0−280, *Citrobacter*: 4−449, and *Serratia*: 0−449) (Table S1), indicating that each species has a large and variable accessory genome.

Since our pan-genome software, PanOCT, does not collapse or discard paralogs, we were able to determine the extent of paralogy within each species’ pan-genome. Of the 37,889 total *E. coli* pan-genome protein clusters, 52% were paralogous, reducing the size of the pan-genome to 18,338 protein clusters, which is still considerably larger than the other species’ pan-genomes (Table 1). *Klebsiella* had the second highest number of paralogs (33%), followed by *Enterobacter*/*Citrobacter* (20% each) and *Serratia* (13%).

A total of 569 CARD/RGI-predicted AMR protein clusters were identified across all four species with Tn-Seq data (i.e., 160 in *E. coli*, 171 in *K. pneumoniae*, 142 in *C. freundii*, and 96 in *S. marcescens*) (Table 1). The distribution of AMR genes across the core and accessory species pan-genomes was roughly even, but with slightly more in the accessory pan-genome (45% core, 55% accessory) except for *S. marcescens* (58% core, 42% accessory). In stark contrast, genes essential for growth under laboratory conditions were skewed toward core (95% core, 5% accessory). Predicted virulence protein clusters varied across species, with *E. coli* and *K. pneumoniae* having more clusters in the accessory pan-genome.

### Fitness genes across species

A total of 695 published bacteremia-fitness genes derived from four Tn-Seq studies (10–13) of *E. coli* (247 genes), *K. pneumoniae* (74 genes), *C. freundii* (172 genes), and *S. marcescens* (202 genes) (Table S2A) were mapped to 662 species pan-genome clusters (Table 1). The distribution of species-specific fitness gene clusters across the core and accessory pan-genomes showed slightly more in the core pan-genome (54% core, 46% accessory), with *K. pneumoniae* (59% core, 41% accessory) and *S. marcescens* (58% core, 42% accessory) each having more bias toward core genes. This indicates that many genes important for causing bacteremia are highly conserved within each species and may be conserved across species as well.

### Multi-species pan-genome

To identify genes conserved across species and determine if they are multi-species bacteremia-fitness factors, core protein sequences from each of the five species-level pan-genomes were combined and analyzed in a second pan-genome run—the MSC pan-genome. For this MSC pan-genome, clusters were labeled as core if they contained proteins from at least four out of five species’ core pan-genomes. There were 2,232 clusters shared across all five species and 2,850 shared in four of five species (Table 1, 100% and 70% cutoff, respectively, and Fig. S1A). *Serratia* had the largest number of species-specific core protein clusters at 1,124, with *Klebsiella* having the second largest number of species-specific core clusters at 892 (Fig. S1A). However, *Escherichia* only had 385 species-specific core protein clusters within the MSC pan-genome.

### Identification of common bacteremia-fitness and virulence genes

Next, we analyzed the MSC pan-genome for bacteremia-fitness genes (Table S3) (see S6 Data set at https://doi.org/10.5281/zenodo.15793688). Using a criterion that at least one member of a MSC pan-genome protein cluster was previously identified by Tn-Seq, 500 clusters were identified as bacteremia-fitness factors, with 422 conserved in four of five species, and 373 were conserved across all five species (Table 1; Fig. S1B). These bacteremia-fitness clusters included proteins from multiple species with published Tn-Seq results implicating them as fitness factors. For example, 11 clusters contained Tn-Seq fitness factors across three species: seven from *C. freundii*, *K. pneumoniae*, and *S. marcescens*, and four from *C. freundii*, *E. coli*, and *S. marcescens* (Table S4). Combining these pan-genome clusters with Tn-Seq data can be used to predict potential fitness genes in species and strains that lack experimentally derived data.

MSC pan-genome clusters with homology to known virulence factors were identified by searching against the virulence factor database (VFDB) (21). Out of 272 total clusters assigned to virulence functions (Table 1), 55 (21%) were conserved across all five species, and 150 were conserved in four or more species (Fig. S1C). The maximum number of shared virulence factors between four, three, and two species was 49 (*Citrobacter:Enterobacter:Escherichia:Serratia*), 12 (*Citrobacter:Enterobacter:Escherichia*), and 7 (*Citrobacter:Escherichia*), respectively. *K. pneumoniae* had the highest number of species-specific virulence factors with 45. We then determined how many MSC pan-genome clusters predicted to be bacteremia-fitness factors were also homologous to known virulence factors (Table S5). Specifically, of the 422 bacteremia-fitness clusters and 150 virulence clusters conserved in four or more species (Table 1; Table S3), 30 were predicted to be bacteremia-fitness factors associated with known virulence mechanisms (e.g., LPS and capsular production for immune modulation, flagella and fimbriae for motility and adherence, and genes for the acquisition of iron and magnesium) (Table S5).

**Fig 2:**
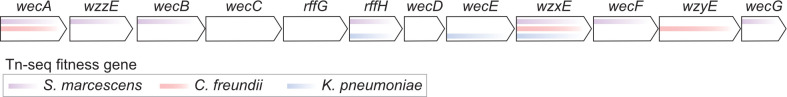
The ECA operon is a shared bacteremia-fitness locus between species. Genes within the conserved ECA operon are represented by arrows, and colored lines indicate genes that were identified as significant bacteremia-fitness genes in previously published Tn-Seq studies.

Using a different approach to identifying multi-species fitness factors, we searched for core operons that contained distinct predicted fitness genes across species. We identified 369 operons that contained at least one bacteremia-fitness factor in the MSC pan-genome (Table S6). Of the 67 operons that contained at least two bacteremia-fitness factors in the MSC pan-genome, 14 operons contained bacteremia-fitness genes from three species, 29 from two species, and 24 from one species (Table S6). One prominent example was the operon encoding the Enterobacterial common antigen (ECA), a 12-gene locus with nine bacteremia-fitness genes identified in either *S. marcescens*, *C. freundii*, or *K. pneumoniae* (Fig. 2). The WzxE ECA flippase within this operon is a predicted fitness factor in all three species, whereas WecA (UDP-*N*-acetylglucosamine-UP *N*-acetylglucosaminephosphotransferase) and RffH (glucose-1-phosphate thymidylyltransferase) proteins were fitness factors in two species.

To prioritize genes for future investigation and identify conserved biological processes contributing to bacteremia, we devised a scoring rubric using four genotypic and phenotypic characteristics (see Materials and Methods for details). The MSC pan-genome bacteremia-fitness genes were individually scored based on the magnitude of their published fitness defect predicted in any bacteremia Tn-Seq screen, whether the same gene was predicted to be a bacteremia-fitness factor in multiple Tn-Seq screens (i.e., multiple species), if multiple bacteremia-fitness factors were encoded in the same operon, and whether mutation of that gene was previously found to increase antibiotic susceptibility of *E. coli* BW25113 (30, 31). Scores were summed in two ways: first, as individual genes to distill the most meaningful bacteremia-fitness factors from the 500 total MSC pan-genome bacteremia-fitness clusters identified, and then as the total score of bacteremia-fitness factors encoded in the same operon to identify biological pathways of interest (Table S7). The rubric produced scores for individual centroids and for operons that most closely mapped to the exponential distribution using the Kolmogorov–Smirnov test with the cutoff capturing the top 10% and 5% of the centroids as shown on the graph (Fig. S2).

Application of this scoring rubric filter led to a reduction of potential bacteremia-fitness factors from 500 to 32 (6.4%) and 73 (14.6%) prioritized bacteremia-fitness factors having centroid scores of >= 8 and >=6, which correspond to centroids in the top 5% and top 10%, respectively (Fig. S2). Additionally, summation of the individual scores for bacteremia-fitness factors encoded within the same operon led to the identification of 11 operons with total scores that were in the top 0.5%. Investigation of the gene functions encoded by these highly scored individual bacteremia-fitness factors and operons revealed seven biological processes that are predicted to contribute to the full virulence capacity of Enterobacterales during bacteremia and could be targets to enhance antibiotic susceptibility (Table 2). These processes include maintenance of the proton motive force, resistance to antimicrobial peptides and complement, protein and small molecule transport, DNA repair and homologous recombination, shikimate biosynthesis, global gene regulation (pre- and post-transcription), and resistance to oxidative stress. Although not an exhaustive accounting of all biological processes that contribute to bacteremia or of the functions encoded within the top scoring genes and operons, these seven biological functions (Table 2) include, at a minimum, 19 of the 32 (59%) fitness genes with scores in the top 5% and 40 of the 73 (55%) fitness genes with scores in the top 10% (Fig. S2). Additionally, these seven biological functions encompass genetic elements encoded by 10 of the 11 highest-scoring bacteremia-fitness factor-rich operons identified.

**TABLE 2:** Core biological processes predicted to be required for full virulence capacity of Enterobacterales during bacteremia using a multifactorial prioritization scoring rubric^*a*^

Function	Gene(s)
Maintenance of proton-motive force across the inner membrane	
ATP synthase	*atpA^‡^B^†^D^†^EG^‡^H^†^*
Electron transport: ubiquinone synthesis	*ubiC^†^GH*
Iron-sulfur cluster biosynthesis for cytochrome maturation	*iscS^‡^U^†^A^†^, hscB^‡^A^†^*
Resistance to antimicrobial peptides and complement	
Modification of lipid A of LPS	*arnAD^‡^E^†^F^†^T*
LPS inner core synthesis	*gmhB^†^*
Synthesis of enterobacterial common antigen	*wecA(rfe)^†^, wzzE, wecB^†^, rffH(rfbA)^‡^, wecE(rffA)^†^, wzxE^‡^, wecF, wzyE, rffM*
Sensitivity to antimicrobial peptides	*sapA^‡^CDF*
Periplasmic protease inactivates complement complex	*prc^†^*
Transport	
Phosphate (ABC) transport	*pstSC^‡^A^‡^, phoU^‡^*
Twin arginine transporter	*tatC^‡^*
DNA repair and homologous recombination	
DNA recombination	*ruvA^‡^BC^‡^*
Tyrosine recombinase	*xerC^‡^D^†^*
Shikimate biosynthesis	
Production of siderophores, aromatic amino acids, folates, and quinones (e.g., *ubiGH*)	*aroACB^†^EK^†^*
Global gene regulation	
Aerobic respiration control protein	*arcAB^†^*
Nucleoid-binding regulator Fis	*fis^†^*
Nitrogen gene regulation sigma factor	*rpoN^‡^*
Glutamine synthetase and glutamine sensing	*glnA^†^D^†^*
mRNA regulation by binding sRNAs to target mRNAs	*proQ^‡^*
Oxidative stress	
Oxidative stress response	*apaGH, pdxA^‡^, subsp.^‡^*

^*a*^ Genes listed here were predicted as a bacteremia-fitness factor in at least one Tn-Seq screen for four species (*E. coli, K. pneumoniae, C. freundii,* and *S. marcescens*) in a murine model of bacteremia and are predicted to contribute to these seven biological processes. Genes were scored among all 500 MSC pan-genome bacteremia-fitness clusters in the four species. Genes followed by *‡* or *†* received total centroid scores ≥8 or ≥6 points, respectively.

### Validation of a representative core fitness gene function for virulence phenotype in a bacteremia mouse model

In our Tn-Seq screens, numerous genes have been predicted to represent fitness factors during experimental bacteremia. This is the first step in identifying relevant genes that are critical for survival in the bloodstream. To validate our findings within the prioritized MSC genes, the twin-arginine translocation (Tat) system was selected for further study. It was selected because of the variable results (i.e., essentiality/fitness roles) from published Tn-Seq studies (10–13) and its role in virulence and antimicrobial susceptibility in other Gram-negative pathogens (11, 32–36) and certain pathotypes of *E. coli* (37, 38). It also tests our operon scoring cutoff since it barely meets the 5% operon score cutoff of 10.28 with a score of 11 (Table S7). The Tat system, under Transport functions (Table 2), facilitates translocation of folded proteins across the cytoplasmic membrane in bacteria, and while the system itself (TatABC) is widely conserved, the translocated proteins that serve as substrates for the system often vary between species (39) and may therefore alter the requirement for the translocation system in different organisms. The *tatC* allele was one of the highest-scoring predicted bacteremia-fitness factors (Table 2; Table S7), and we previously validated it as a fitness gene in *C. freundii* (11).

**Fig 3:**
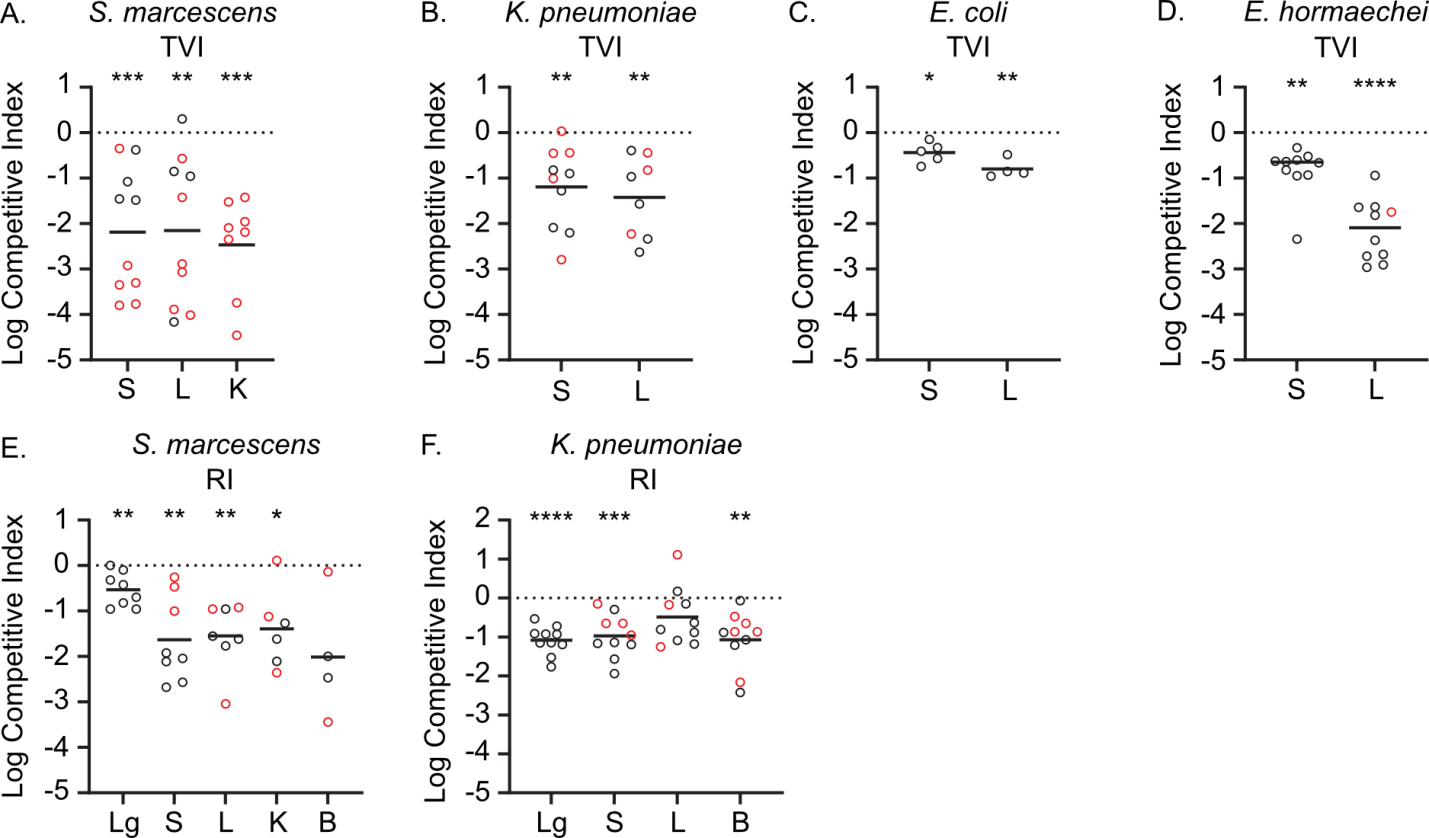
Validation of *tatC* contributions to bacteremia fitness. Mice were inoculated via tail vein injection (TVI) or retropharyngeal inoculation (RI) with a mixture of wild-type and *tatC* mutant derivatives of *S. marcescens* (**A and E**), *K. pneumoniae* (**B and F**), *E. coli* (**C**), or *E. hormaechei* (**D**). Viable bacteria were enumerated in the spleen (S), liver (L), kidneys (K), lungs (Lg), or blood (B) at 24 h post-inoculation, and the relative fitness of each strain was determined as the log competitive index. Only *S. marcescens* was assayed in the kidneys based on previously determined infection profiles (8). Mean indices (solid line) that deviated significantly from the hypothetical value of zero (dotted line), which represents neutral fitness, were determined by one-sample *t*-test: * (*P* < 0.05), ** (*P* < 0.01), *** (*P* < 0.001), **** (*P* < 0.0001). Red symbols designate samples from which *tatC* mutant bacteria were at or below the limit of detection.

To directly test whether TatC was a conserved bacteremia-fitness factor among the remaining four species of interest, *tatC* mutants were constructed in *S. marcescens*, *K. pneumoniae, E. hormaechei,* and *E. coli,* followed by competition infections whereby the wild-type strain and the differentially marked *tatC* mutant strain were co-inoculated in a murine infection model. For all four tested species, the *tatC* mutant was significantly outcompeted by the wild-type strain when bacteria were inoculated into the bloodstream via tail vein injection (TVI), as indicated by competitive indices that were significantly below zero (Fig. 3A through D). Furthermore, *tatC* was required in every tested organ via this model, demonstrating that folded protein translocation was universally important not only across species but within different infection niches.

Since the TVI route represents direct bacteremia, we additionally sought to determine how TatC contributes to disseminated infection via a bacteremic pneumonia model. Following retropharyngeal inoculation (RI), *S. marcescens* and *K. pneumoniae tatC* mutants were outcompeted in the lungs of infected mice (Fig. 3E and F). For *S. marcescens*, an even greater competitive disadvantage was observed for bacteria that had escaped the primary infection site and disseminated to the spleen, liver, and kidneys (Fig. 3A and E). This result was further supported by a trend toward lower recovery of the *tatC* mutant from the bloodstream and suggests that Tat translocation is critically important for escape from the lungs or survival in distal sites for this organism. Similarly, *K. pneumoniae tatC* mutants that disseminated from the primary infection site were also significantly outcompeted by wild-type bacteria (Fig. 3B and F). Together, these results establish the importance of the Tat translocation system, and very likely its substrate proteins, in Gram-negative bacteremia and demonstrate the value of comparing Tn-Seq fitness hits between species.

### Contribution of conserved fitness genes to intrinsic antibiotic resistance

**Fig 4:**
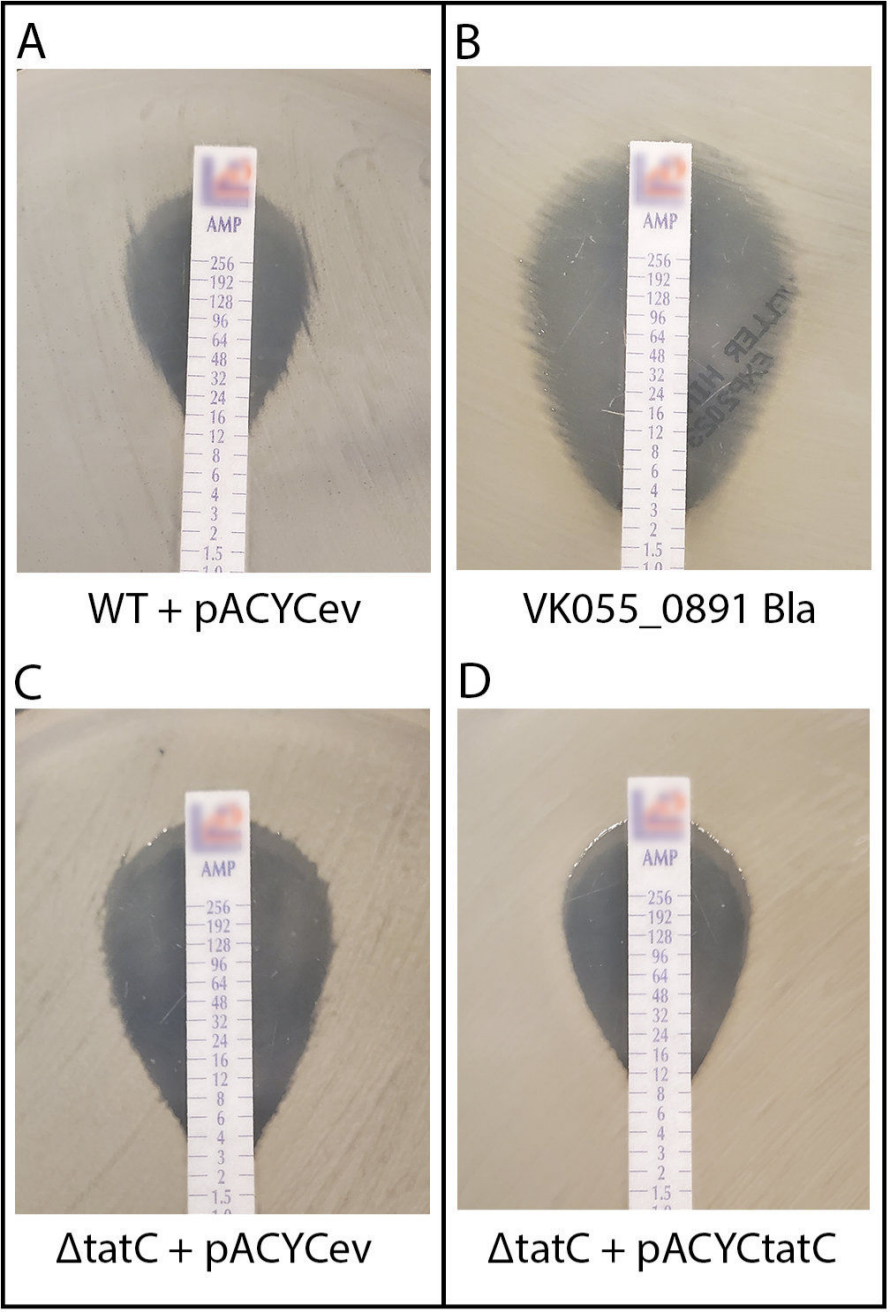
Disruption of *K. pneumoniae tatC* confers susceptibility to ampicillin. Epsilometer (E-test) results for ampicillin are shown with minimum inhibitory concentration (MIC) at the point the zone of clearing touches the test strip for (**A**) WT KPPR1 + pACYCev (pACYC184), (**B**) KPPR1 *bla* transposon mutant VK055_0891_bla, (**C**) KPPR1 Δ*tatC* +pACYCev, and (**D**) KPPR1 Δ*tatC* + pACYC*tatC*.

Previous work in *E. coli* revealed that many core genes contributed to antibiotic resistance that were not predictable based on their known functions (30, 31). Genes that contribute to both bacteremia fitness and antibiotic resistance could be attractive therapeutic targets, as disruption of their function in combination with antibiotic therapy would be expected to have synergistic efficacy. Therefore, we mapped published data on the antibiotic susceptibility of a comprehensive panel of *E. coli* mutants to multiple classes of antibiotics to our MSC pan-genome. *E. coli* mutants in *tatC* were previously shown to have increased susceptibility to β-lactam antibiotics. Unlike *E. coli*, the species *K. pneumoniae*, *S. marcescens*, *E. hormaechei,* and *C. freundii* have intrinsic resistance to ampicillin due to chromosomally encoded β-lactamases. To test whether disruption of *tatC* by either a transposon insertion (*tatC::tn*) or deletion (Δ*tatC::nptII*) could counteract this intrinsic resistance, we used Epsilometer tests to determine the minimum inhibitory concentration (MIC) of ampicillin or piperacillin-tazobactam for the wild-type and *tatC* mutants of these four species. The most striking finding was in *K. pneumoniae*, where disruption of *tatC* by either approach lowered the MIC to <8 µg/mL, which is a level that would be considered susceptible to ampicillin in clinical care of an infection and is similar to disruption of the β-lactamase itself (*bla::tn*) (Table 3; Fig. 4). This increased susceptibility to ampicillin in *K. pneumoniae* was counteracted by provision of either *tatC* (pACYC*tatC*) or the *tat* operon (pACYC*tatABCD*) on a multicopy plasmid, but not the empty vector control (pACYCev). When *tatC* was interrupted in a carbapenem-resistant isolate of *E. hormaechei*, the resistance profile shifted from resistant to sensitive dose dependent (SDD) to piperacillin-tazobactam (Table 3). The resistance phenotype of the *tatC* mutant was returned to parental levels when *tatABC* was provided on a plasmid. In the case of *S. marcescens*, which has an extremely high resistance to ampicillin (> 256 µg/mL), mutation of *tatC* caused a substantial reduction in MIC, which could be rescued by provision of the *tatC* in *trans* from a multicopy plasmid (pBB26) and not the vector control (pBBR1MCS-5) (Table 3). In *C. freundii*, we tested piperacillin-tazobactam as a β-lactam and β-lactamase inhibitor combination without intrinsic resistance and found that a *tatC* mutation also lowered the MIC, and this phenotype was likewise rescued by provision of *tatC* (pBB4 (*tatC*+)) in *trans* and not the vector control (pBBR1MCS-5; Table 3). Combined with animal models of infection, these data demonstrate that *tatC* represents a conserved bacteremia-fitness factor that also contributes to intrinsic resistance to β-lactam antibiotics in Enterobacterales, and the disruption of TatC function has the potential to restore the use of antibiotics to which these species have intrinsic or acquired resistance.

**TABLE 3:** Susceptibility of *tatC* mutants to β-lactam antibiotics

Species (strain)	Genotype	Antibiotic	Mean MIC (µg/mL)	CLSI interpretation^*a*^
*K. pneumoniae* (KPPR1)	Wild-type	Ampicillin	24.0	I
	Wild-type + pACYCev		18.7	I
	*tatC*::tn		8.0	S
	*tatC*::*nptII*		6.7	S
	*bla*::*tn*		3.0	S
	*tatC*::tn + pACYCev		6.7	S
	*tatC*::tn + pACYC*tatC*		14.7	I
	*tatC*::tn + pACYC*tatABCD*		18.7	I
*S. marcescens* (UMH9)	Wild-type	Ampicillin	>256	R
	*tatC*::*nptII* + pBBR1MCS-5		85.3	R
	*tatC*::*nptII* + pBB26 (*tatC*+)		>256	R
*C. freundii* (UMH14)	Wild-type	Piperacillin-tazobactam	6	S
	*tatC*::*nptII* + pBBR1MCS-5		0.75	S
	*tatC*::*nptII* + pBB4 (*tatC*+)		5.3	S
*E. hormaechei* (CRE14)	Wild-type	Piperacillin-tazobactam	96	R
	*tatC*::*acc (3)IV* + pBBR1Hyg		16	SDD
	*tatC*::*acc (3)IV* + pBBR1Hyg_*tatABC*		85.3	R

^*a*^ S, sensitive; SDD, sensitive dose dependent; I, intermediate; R, resistant.

## DISCUSSION

Although Tn-Seq has become a powerful tool to simultaneously assess the contribution of all non-essential genes in animal models of infection, mechanistic studies for the evaluation of specific genes in animal models of bacteremia have been conducted only sparingly (40). Although there have been more than 200 published Tn-Seq studies dealing with bacterial pathogens *in vitro* or *in vivo* (12, 41–91), only a fraction of these have addressed Gram-negative pathogens in animal models of infection (12, 41, 43, 49, 53, 55, 58, 65, 67, 68, 72, 76, 78–91). In practice, characteristics of Gram-negative bacterial species causing bacteremia have been hard to identify, and only a few studies (65, 92, 93), beyond our work (10, 12, 13, 80, 94–96), have sought fitness and virulence factors in models of bacteremia. Despite the valuable insight Tn-Seq studies have provided at the organismal level, comprehensive information on shared and unique fitness strategies across Gram-negative species is lacking, due in part to the unique nature and depth of each transposon insertion library and a lack of computational tools to compare multiple data sets across pan-genomes.

Because each of our published bacteremia Tn-Seq studies (10–13) seemed to identify different genes (i.e., no single gene was found to be associated with bacteremia-fitness across all studies/organisms), we were unable to identify operons/pathway associations until we took our computational data integration approach presented here. In this report, we leveraged the JCVI pan-genome pipeline as a tool to integrate multiple disparate data types to identify key pathways and mechanisms used by Enterobacterales to survive in the bloodstream. Within the core genes (2,850/2,232, 70%/100% core, respectively) shared by *E. coli*/*Shigella spp., K. pneumoniae, S. marcescens*, *C. freundii,* and *E. hormaechei,* statistically significant bacteremia-fitness factors were identified in core clusters (424/373, 70%/100% core, respectively) containing at least one bacteremia-fitness factor identified in any of the four Tn-Seq studies performed in our TVI model of bacteremia. Using an objective scoring rubric, we propose prioritization of 73 of the 500 total conserved bacteremia-fitness factors found in the MSC pan-genome that have scores in the top 10% for future investigation. Of these 73 prioritized fitness factor genes, 36 were predicted by Tn-Seq to contribute to bacteremia in at least two species, and 41 were found to increase antibiotic susceptibility in *E. coli* BW25113 when mutated (30, 31). Summation of individual gene scores encoded in the same operon revealed 27 operons enriched for bacteremia-fitness factors with scores in the top 5%. Investigation of the biological functions encoded by these operons and high-scoring bacteremia fitness factors revealed a minimum of seven common biological functions that significantly contribute to Enterobacterales fitness during bacteremia. These data support a model in which the full virulence of Enterobacterales during bacteremia, in part, requires the seven biological functions outlined in Table 2 and discussed below.

To aid in the discovery of potential novel antibiotic targets to clear bacteremia, we conducted species-level and order-level pan-genomic analyses that identified the core pathogenic genome of these Enterobacterales species. The identification of unique and common virulence determinants among these Gram-negative bacterial pathogens also provided substantial advances toward determining mechanisms of pathogenesis for these pathogens that disseminate to and survive in the bloodstream. By integrating the pan-Enterobacterales genome, genome-wide Tn-Seq fitness data (10–13) and published lists of essential genes (23–27), antibiotic mutational susceptibility (30, 31), virulence (21), and operon data (28, 29), we have identified 373 conserved fitness genes across all five species that were shown to be important in at least one species during experimental bacteremia. Inhibition of these bacteremia-fitness factors *in vivo* could allow the immune system to clear infecting bacteria more rapidly or even kill them directly. Furthermore, 95 mutants in these bacteremia-fitness genes also confer increased susceptibility to common antibiotics in a laboratory strain of *E. coli* (30, 31). This raises the exciting possibility that inhibitors of these gene products can simultaneously decrease *in vivo* fitness and sensitize the bacteria to FDA-approved standard-of-care antibiotics.

The MSC pan-genome produced 500 orthologous protein clusters that contained at least one bacteremia-fitness factor from at least one published Tn-Seq data set, which is too many to validate. Thus, we devised an objective scoring rubric that combined four lines of pro-fitness biologically meaningful evidence (e.g., the published fold-change of the fitness defect associated with transposon interruption of a gene in any one species, being identified as a fitness factor in multiple species, whether multiple fitness factors were encoded in the same operon, and if previously found to confer increased antibiotic susceptibility) as a non-statistical way to filter the list of 500 potential bacteremia fitness genes to a reduced and manageable set of 32 genes to independently knock out in each pathogen and test in the murine model of bacteremia. This resulted in several conserved and shared Enterobacterales genes that fall into common bacterial cell functions, of which we discuss seven (Table 2). These included (i) the maintenance of proton-motive force across the inner membrane, (ii) resistance to antimicrobial peptides and complement, (iii) substrate transport, (iv) genome maintenance, (v) shikimate biosynthesis, (vi) global gene regulation, and (vii) oxidative stress. Although it is beyond the scope of this study to discuss all these findings, it is instructive to summarize notable predicted bacteremia-fitness factors below in the context of the seven highlighted common bacterial cell processes that support survival of Enterobacterales species in the mammalian bloodstream.

Enterobacterial common antigen is a critical outer membrane component in Enterobacterales that may affect resistance to antimicrobial peptides and complement (97). Interestingly, although identified by Tn-Seq in *K. pneumoniae*, *C. freundii*, and *S. marcescens* (Fig. 2), none of the ECA genes were identified by Tn-Seq as fitness factors in *E. coli,* suggesting that either the biological role of ECA during infection differs in this species or that ECA genes were not adequately targeted by transposon mutagenesis in *E. coli*. Although this discrepancy is currently under investigation, the clear benefit of such a comparison is in forming testable hypotheses regarding the conserved function of ECA or other shared operons across Gram-negative bacteremia-causing species. Combined, our findings provide strong evidence that production of ECA is an important fitness determinant since mutation of even a single gene in the processive ECA biosynthesis pathway may result in a lack of ECA production or its proper localization in the bacterium. Clearly, surface-exposed macromolecules on the Gram-negative outer membrane, when present or absent or biochemically modified, may modulate surface charge. These changes in charge can confer, in some cases, resistance to binding by complement components and host-derived antimicrobial peptides, thus conferring protection from these host innate immune responses.

Twin-arginine translocation system (98) represents perhaps the most critical transport system and fitness factor overall required for bacteremia. The TatC transmembrane protein was previously shown to contribute to bacteremia fitness in *C. freundii* (11) and *K. pneumoniae* (13) (Table S2A). However, neither the *S. marcescens* (10) nor *E. coli* (12) Tn-Seq findings identified TatC as a significant fitness factor, and it was even found to be essential in *S. marcescens* for growth on laboratory media (20). Tat mutants of *Salmonella enterica* serovar Typhimurium displayed increased susceptibility to antibiotics that target the cell wall (99), as did a laboratory strain of *E. coli* (30). The Tat secretion was also shown to have a role in virulence phenotypes in other Gram-negative pathogens (11, 32–36) and two pathotypes (e.g., EHEC and ExPEC) of *E. coli* (37, 38). It was this discrepancy in essentiality and roles in antibiotic susceptibility and virulence, in addition to the relatively low operon score of 11 (i.e., a score of 10.28 is at the 5% cutoff), that motivated our decision to test independent *tatC* knockouts in *K. pneumoniae*, *S. marcescens*, *E. coli,* and *E. hormaechei* for essentiality, fitness, and antibiotic susceptibility. Despite this apparent requirement for *tatC*, it is intriguing that we were able to produce viable and stable independent deletions of *tatC* in the four species studied here, suggesting that *tatC* is not essential for growth. Perhaps the discrepancy with previous studies is due to the lack of sufficient representation of *tatC* mutants in the inoculum or the initial transposon library, increased susceptibility to the antibiotic used in genetic screens, or strain-specific differences in Tat requirements. Our results showed that the *tatC* allele demonstrated a significant contribution to the fitness of all four species tested in this study in a murine model of bacteremia (Fig. 3). Although it is possible that disruption of the TatC transmembrane component may have unintended consequences, such as altering bacterial envelope integrity, the most likely explanation for this shared fitness phenotype is a requirement for appropriate localization of Tat-secreted proteins for infection fitness. Indeed, we have previously attributed at least some of the fitness defects associated with *C. freundii tatC* mutation to SufI loss of function (11). Whether SufI is required for infection of these additional species remains to be determined. However, it may also be informative in future work to compare the repertoire of Tat-secreted proteins in each species using the conserved N-terminal twin-arginine motif, since it is additionally possible that the requirement for TatC is due to species-specific translocation of different proteins.

### Conclusions

The use of our pan-genome pipeline as a powerful tool for integrating multiple data types and sources to infer biological functions was demonstrated in this study. Comparison of the four Enterobacterales Tn-Seq data sets revealed, at first inspection, limited common mutations and functional insight. It was not until those results were mapped to orthologous protein clusters and overlaid upon operon structure that common pathways emerged (Table 2). This enabled the prediction of common genes within this core pan-genome and brought to light mechanisms that are essential for colonization of, or survival in, the mammalian bloodstream. This represents a step forward in the quest to identify novel targets of therapy against these deadly, widespread infections often accompanied by the development of life-threatening sepsis. Our prediction and subsequent validation of *tatC* as a common bacteremia fitness factor (Fig. 3), and further demonstration of increased antibiotic susceptibility of strains carrying mutations in this allele (Fig. 4; Table 3), support the utility of our bioinformatic method for integration and prioritizing of other biological data sets to identify genes and pathways of interest. Having predicted the importance of many more genes (Table S7) and pathways, highlighted in Table 2, we have gone on to validate nine additional multi-species fitness factors in our murine model of bacteremia (100). This Enterobacterales multi-species core genome can be used as an atlas for identifying common mechanisms and therapeutic targets in these common and increasingly antibiotic-resistant pathogens.

## MATERIALS AND METHODS

### Species-level pan-genome analysis

The pan-genome for the five Enterobacterales species was constructed using all the proteins from a curated list of publicly available genomes for each species (Table S1) using the JCVI pan-genome pipeline (14). Specifically, we used the “user_core_cutoff” branch of the JCVI pan-genome pipeline found on GitHub (101). These included the reference genomes used for Tn-Seq as well as quality genomes from UMH, common laboratory strains, and isolates of epidemiological importance. The number of genomes in each species-level pan-genome run ranged from a minimum of 28 (*Citrobacter*) to a maximum of 281 (*Escherichia*) and consisted mainly of a single species per genus (e.g., *C. freundii*, *E. hormaechei, K. pneumoniae,* and *Serratia marcescens*), where only the *Escherichia* pan-genome also contained *Shigella* spp. The default parameters for the pan-genome pipeline were used, except for a core cutoff (i.e., run_pangenome.pl --core_cutoff) of 70% of genomes as opposed to the 95% default.

### Enterobacterales multi-species core (MSC) pan-genome analysis

The pan-genome pipeline results were used to create a core “pseudo-genome” for each species from the sequences of the core centroids and their location on the species-level consensus pan-chromosome, as shown in the Core.att attribute output file (102). These core pseudo-genomes from the five species were integrated into a MSC pan-genome using the same parameters, including the 70% core cutoff. A consensus MSC pan-chromosome was constructed as described for the species-level pan-chromosomes. For all species’ pan-genomes, and the MSC pan-genome, the TIGR roles and AMR were assigned to centroids using the best match for the HMMer3 (103) alignment of the TIGRFAMs (104) and the CARD (22), respectively, as part of the pan-genome pipeline (14). Other high-level gene features were integrated into the pan-genomes as described below.

### Integration of Tn-Seq bacteremia-fitness genes with pan-genomes

As the original Tn-Seq fitness experiments were performed on single bacterial strains (10–13) whose genomes were included in the individual species pan-genomes (e.g., *C. freundii* UMH14 biosample SAMN07729546, UPEC *E. coli* CFT073 biosample SAMN02604094, *K. pneumoniae* ATCC 43816 KPPR1 biosample SAMN02982872, and *S. marcescens* UMH9 biosample SAMN06164063), we were able to annotate the fitness gene clusters using their unique gene identifiers (Table S2A). If the species pan-genome bacteremia-fitness clusters were core (i.e., orthologous sequences present in greater than 70% of the genomes), they were used in the MSC pan-genome and the MSC pan-genome clusters were labeled a fitness centroid.

### Integration of operon structures, essential and virulence factors, and KEGG ontology

Experimentally determined operon structures for *E. coli* and *Klebsiella* (28, 29, 105) were obtained and mapped to the MSC pan-genome centroids with BLASTP (reciprocal best matches with E-value <1 × 10^−5^). For the *E. coli* operons, additional information, such as the Gene Symbol and the Blattner identifier, was also included (Table S3). To include as many Blattner identifiers as possible for the MSC pan-genome clusters, in instances where the MSC clusters were not in operons, we used the Blattner identifier and the gene symbol of any gene from the genome of *E. coli* str. K-12 substr. MG1655 (BioSample SAMN02604091) with a good BLASTP match (same cutoff as above) to the MSC pan-genome clusters. We also mapped the antibiotic susceptibility of the genes from (30, 31) using the Blattner-derived gene symbols.

Genes that were found to be essential for the growth of *E. coli* K-12 and derivatives under laboratory growth conditions were obtained from five different manuscripts/databases (23–27). A unique set of “essential” genes was created (Table S2B) from these combined data sets and assigned the unique *E. coli* K-12 composite (ECK*) identifier. BLASTP was used to map the translated essential proteins to the *Escherichia* pan-genome centroids.

Similarly, we used the full set of protein sequences from the Virulence Factor Database (VFDB) (Table S2C) (21) downloaded on August 31, 2021, to do a similar BLASTP mapping to species-level pan-genome centroids. If the reciprocal best matches had an E-value <1 × 10^−5^ and an additional requirement that the BLASTP match had to have more than 70% identity, then the centroid was annotated as “Essential” or a “Virulence Factor.” The cluster annotation for the species-level pan-genome was passed to the MSC pan-genomes as described for bacteremia-fitness proteins above.

Additionally, KEGG annotations were mapped to the MSC centroids using hmmsearch v 3.3.2 (106) against the KEGG ontology data set collected in KOfam (107) with an E-value <1 × 10^−5^ (Table S3).

### Venn diagrams

Venn diagrams were created with R with the Venn Package (108), using data from Table S3. For each of the Venn diagrams comprising Fig. S1**,** a different list of MSC cluster IDs was used as input for each species in the MSC pan-genome to count the numbers of shared orthologs in: (i) all MSC clusters, (ii) all MSC clusters with at least one Tn-Seq bacteremia-fitness protein, and (iii) all MSC clusters with at least one virulence factor protein.

### Pan-chromosome visualizations

Browsable pan-chromosome visualizations, including the fitness, antimicrobial resistance, and TIGRFam annotations, were constructed for each species pan-genome and the MSC pan-genome with PanACEA using default parameters (see S1–S5 Datasets at https://doi.org/10.5281/zenodo.15793688) (Table S3) (18). The species pan-genome comparison was constructed with data from Table S3, using a polar graph from ggplot2 (109). Orthologs for each MSC cluster were identified in each species, as well as the relative position in each species’ pan-genome relative to *dnaA*. MSC clusters that were not present in a species were noted with a gap. The radial position of orthologs on the figure was the relative location of the *Escherichia* ortholog, whereas the color was based on the location of the ortholog in each species. The fitness clusters with an *Escherichia* ortholog are shown at the *Escherichia* ortholog position.

### Prioritization of fitness genes and operons

We ranked MSC pan-genome bacteremia-fitness clusters and operons identified among the four species Tn-Seq data using an objective scoring rubric. The rubric was based on the following four additive criteria: (i) the published fold-change of the fitness defect associated with transposon interruption of a gene in any one species, (ii) a single gene being identified as a fitness factor in multiple species, (iii) whether multiple fitness factors were encoded in the same operon, and (iv) a fitness factor single-gene-knockout was previously found to confer increased antibiotic susceptibility in *E. coli* BW25113 (30, 31).

The top 60 bacteremia-fitness factors in any one species Tn-Seq data set (i.e., the top 60 least fit transposon mutants) were awarded points based on the published magnitude of the fitness defect determined in any one species. The top 1−20 least fit bacteremia-fitness factors were awarded three points, 21−40 were awarded two points, and 41−60 were awarded one point. All scores for each MSC bacteremia-fitness factor were then summed across species (e.g., *wzxE* received six total points in this category by accumulating three points each for providing the 6th and 15th greatest fitness defects when mutated in *S. marcescen*s UMH9 and *K. pneumoniae* KPPR1, respectively.) One item of note, *K. pneumoniae* KPPR1 bacteremia Tn-Seq (13), only identified 55 fitness factors; therefore, this species was slightly underrepresented in this scoring category.

The MSC pan-genome bacteremia-fitness factor clusters were also awarded points based on their identification in multiple species Tn-Seq data sets, regardless of the magnitude of their defect. If a cluster member was identified as a bacteremia-fitness factor in three species or two species, it was awarded three points or two points, respectively. (e.g., *wzxE* received three points for being identified as a bacteremia-fitness factor in Tn-Seq data sets from *C. freundii* UMH14, S. *marcescen*s UMH9, and *K. pneumoniae* KPPR1.)

The MSC pan-genome bacteremia-fitness factor clusters were awarded points based on the identification of at least one other bacteremia-fitness factor encoded in the same operon, regardless of which species any one fitness factor was identified in. Fitness factors found encoded in an operon with 5−9 other fitness factors were awarded three points, those encoded with 3−4 other fitness factors were awarded two points, and those encoded in an operon with 1−2 other fitness factors were awarded one point. An additional point was awarded to fitness factors found in an operon where at least 50% of the genes in that operon were found to be fitness factors. (e.g., *wzxE* is encoded in a 12-gene operon with nine other fitness factors being identified in at least one species’ Tn-Seq data set. Therefore, *wzxE* received a total of 4 points; three points for being encoded in an operon with 5−9 other fitness factors and one additional point for greater than 50% of the genes in that operon being identified as fitness factors.)

Finally, MSC pan-genome bacteremia-fitness factor clusters were given additional points if mutation of that gene in *E. coli* BW25113 conferred increased susceptibility to an antibiotic (30, 31). Fitness factor clusters received another two points if mutation of the orthologous gene in the Keio collection was found to have increased sensitivity to any antibiotics, regardless of the mode of action.

A histogram of the total score of both individual centroids and operons was plotted with ggplot2 (109), and the Kolmogorov–Smirnov test, in the stats library in R (110), was used to determine the most representative distribution. The most representative distribution (exponential) was used to find the cutoff values, giving the top-scoring 5% and 10% of the centroids and operons.

### Generation of the *tatC* mutant derivatives and complementation constructs

Mutations in *tatC* were generated by lambda red recombineering using established protocols (10, 11, 80, 111). For *C. freundii* (constructed previously, [11]), *E. coli, K. pneumoniae,* and *S. marcescens*, the *nptII* kanamycin resistance cassette was PCR-amplified from pKD4 (111, 112) and targeted to an in-frame deletion of the *tatC* ORF via 5’-end homologous sequences (Table S8). For *E. hormaechei,* the *acc (3)IV* apramycin resistance cassette was amplified from pUC18-miniTn7T-Apr (113). Recombination was facilitated by Red functions encoded on pKD46, pSIM19, or pSIM18, depending on the species mutated (111, 112). All mutations were confirmed by sequencing of PCR-amplified alleles or by PCR amplicon size, and recombineering plasmids were cured prior to phenotypic analysis.

Genetic complementation of the *S. marcescens tatC* mutation was achieved by insertion of the UMH9 *tatC* ORF into plasmid vector pBBR1MCS-5 (114) via isothermal assembly, followed by sequencing verification. The resulting plasmid, pBB26, was transformed into *S. marcescens* via electroporation. Genetic complementation of the *K. pneumoniae tatC* mutation with the pAYCYC_tatC_ plasmid was achieved by using SOE PCR (115) to connect the promoter of the *tat* operon to the *tatC* ORF. The pACYC_tatABCD_ plasmid insert was made by amplifying *tatABCD,* including its native promoter. The amplicons were inserted into pACYC184 at the *Hin*dIII and *Sal*I sites, and the resulting plasmids were sequenced for verification. Correct plasmids were transformed into *K. pneumoniae* via electroporation. The *E. hormaechei tatC* mutant was complemented by insertion of wild-type *tatABC* into pBBR1Hyg, a hygromycin-resistant derivative of pBBR1MCS-5 (this study), by isothermal assembly. The resulting plasmid, pBBR1Hyg_*tatABC,* was transformed into *E. hormaechei* by electroporation.

### Murine bacteremia models

Wild-type and *tatC* mutant derivatives were cultured separately in LB medium, then washed with PBS and normalized to the final density prior to mixing 1:1. Tail vein injections were performed on 7- to 8-week-old C57BL/6 J mice as previously described for all tested species (9, 40), and organs were collected for homogenization and CFU determination at 24 h post-injection. For the bacteremic pneumonia model, 1 × 10^7^ CFU *S*. *marcescens* or 1 × 10^6^ CFU *K*. *pneumoniae* was administered retropharyngeally into lightly anesthetized mice, as previously described (80). Mice were sacrificed at 24 h post-inoculation for CFU determinations. Competitive indices for both infection models were determined as the ratio of mutant/wild-type bacteria after 24 h compared with that same ratio in the inoculum.

### Determination of minimum inhibitory concentration

The minimum inhibitory concentration (MIC) of ampicillin and piperacillin-tazobactam was determined using Epsilometer (E-test) strips. This experiment was carried out following the guidelines of the Clinical Microbiology Procedure Handbook (116). Bacteria were cultured on Lysogeny Broth (LB) agar, then inoculated in 3 mL of LB broth the following day. After reaching an OD_600_ of 0.1, cultures were spread onto Mueller-Hinton agar using a sterile cotton swab and allowed to dry. Then, the E-test strips were placed on the dried plates and incubated at 37°C overnight. The MIC was determined by observing the point at which the zone of clearing touches the test strip. The Clinical and Laboratory Standards Institute (CLSI) interpretations were obtained from *Performance Standards for Antimicrobial Susceptibility Testing* (117).

## SUPPLEMENTAL MATERIAL

Fig. S1 mbio.01988-25-s0001.docx Proteins shared across five bacteremia-causing Enterobacterales species in the MSC pan-genome.

Fig. S2 mbio.01988-25-s0002.docx Distribution of bacteremia-fitness factor centroid scores.

Table S1 mbio.01988-25-s0003.xlsx Genomes used in this study.

Table S2 mbio.01988-25-s0004.xlsx Tn-Seq fitness genes, *E. coli* essential genes, and virulence genes used in this study.

Table S3 mbio.01988-25-s0005.xlsx Multi-species core pan-genome clusters.

Table S4 mbio.01988-25-s0006.xlsx Tn-Seq fitness factors shared across at least 3 species.

Table S5 mbio.01988-25-s0007.xlsx Bacteremia-fitness and virulence MSC clusters.

Table S6 mbio.01988-25-s0008.xlsx Fitness genes mapped to known operons.

Table S7 mbio.01988-25-s0009.xlsx Ranking of fitness genes.

Table S8 mbio.01988-25-s0010.xlsx Oligonucleotide primers used in this study.

ASM does not own the copyrights to Supplemental Material that may be linked to, or accessed through, an article. The authors have granted ASM a non-exclusive, world-wide license to publish the Supplemental Material files. Please contact the corresponding author directly for reuse.
