# Supplementary material for: Integrating genomic and Tn-Seq data to identify common in vivo fitness mechanisms across multiple bacterial species
Source: mBio. 2025 Sep 22;16(11):e01988-25. doi: 10.1128/mbio.01988-25 (PMC12607656; doi:10.1128/mbio.01988-25)
Supplement: Fig. S1 — Proteins shared across five bacteremia-causing Enterobacterales species in the MSC pan-genome. [file mbio.01988-25-s0001.docx]

**S1 Fig. Proteins Shared Across Five bacteremia-causing *Enterobacterales species* in the MSC Pan-genome.**  All orthologous protein clusters (**A**), the clusters labeled as “bacteremia-fitness” from Tn-Seq data (**B**), and the clusters labeled as “virulence factor” with matches to the VFDB (**C**) from the MSC pan-genome were binned, counted, and placed into a Venn diagram by whether clusters contained proteins from *C. freundii*, *E. hormaechei,* *E. coli/Shigella* spp., *K. pneumoniae*, and *S. marcescens*. Singleton clusters, representing species-specific core genes, are noted in the outermost ellipses of the Venn diagram. The Venn diagram is not to scale.
