# Supplementary material for: Integrating genomic and Tn-Seq data to identify common in vivo fitness mechanisms across multiple bacterial species
Source: mBio. 2025 Sep 22;16(11):e01988-25. doi: 10.1128/mbio.01988-25 (PMC12607656; doi:10.1128/mbio.01988-25)
Supplement: Fig. S2 — Distribution of bacteremia-fitness factor centroid scores. [file mbio.01988-25-s0002.docx]

**S2 Fig. Distribution of Bacteremia-fitness factor centroid scores.** Summary of total centroid and total operon scores for all 500 bacteremia-fitness factors (**A**) and 366 bacteremia-fitness operons (**B**) scored in S9 Table. The vertical black dotted lines depict the top 5% cutoff and the gray vertical dotted lines depict the top 10% cutoff using the exponential distribution. Light gray box in (**A**) indicates the 73 bacteremia-fitness factors with a total centroid score in the top 10% of the scores (i.e., genes receiving at least 6 total points, and includes both † and ‡ genes highlighted in Table 2). The dark gray box indicates the 32 bacteremia-fitness factors with a total centroid score in the top 5% (i.e., genes receiving at least 8 total points, including only ‡ genes highlighted in **Table 2**). The colored lines show the different distributions that were compared to the scores, including the exponential (red), poisson (blue) and Gaussian (purple). The Kolmogorov-Smirnov test indicated that the Exponential Distribution was the closest fit and was used.
